# Elevated CD169 expressing monocyte/macrophage promotes systemic inflammation and disease progression in cirrhosis

**DOI:** 10.1007/s10238-024-01305-3

**Published:** 2024-02-28

**Authors:** Lichen Xu, Chunhong Huang, Xiaoping Zheng, Hainv Gao, Sainan Zhang, Mengfei Zhu, Xiahong Dai, Gang Wang, Jie Wang, Haolu Chen, Haihong Zhu, Zhi Chen

**Affiliations:** 1 Department of Nephrology, Sir Run Run Shaw Hospital, Zhejiang University School of Medicine, Hangzhou, People’s Republic of China; 2 Department of Clinical Laboratory, The Second Affiliated Hospital, Zhejiang University School of Medicine, Hangzhou, People’s Republic of China; 3 Department of Pathology, Shulan (Hangzhou) Hospital Affiliated to Zhejiang Shuren University Shulan International Medical College, Hangzhou, People’s Republic of China; 4 Department of Infectious Diseases, Shulan (Hangzhou) Hospital Affiliated to Zhejiang Shuren University Shulan International Medical College, Hangzhou, People’s Republic of China; 5 Shulan International Medical College, Zhejiang Shuren University, Hangzhou, People’s Republic of China; 6 State Key Laboratory for Diagnosis and Treatment of Infectious Diseases, National Clinical Research Center for Infectious Diseases, Collaborative Innovation Center for Diagnosis and Treatment of Infectious Disease, The First Affiliated Hospital, Zhejiang University School of Medicine, 79 Qingchun Road, Hangzhou, 310003 Zhejiang People’s Republic of China

**Keywords:** Cirrhosis, Systemic inflammation, CD169

## Abstract

Systemic inflammation is related to disease progression and prognosis in patients with advanced cirrhosis. However, the mechanisms underlying the initiation of inflammation are still not fully understood. The role of CD169^+^ monocyte/macrophage in cirrhotic systemic inflammation was undetected. Flow cytometry analysis was used to detect the percentage and phenotypes of CD169^+^ monocytes as well as their proinflammatory function in patient-derived cirrhotic tissue and blood. Transcriptome differences between CD169^+^ and CD169^−^ monocytes were also compared. Additionally, a mouse model with specific depletion of CD169^+^ monocytes/macrophages was utilized to define their role in liver injury and fibrosis. We observed increased CD169 expression in monocytes from cirrhotic patients, which was correlated with inflammatory cytokine production and disease progression. CD169^+^ monocytes simultaneously highly expressed M1- and M2-like markers and presented immune-activated profiles. We also proved that CD169^+^ monocytes robustly prevented neutrophil apoptosis. Depletion of CD169^+^ monocytes/macrophages significantly inhibited inflammation and liver necrosis in acute liver injury, but the spontaneous fibrin resolution after repeated liver injury was impaired. Our results indicate that CD169 defines a subset of inflammation-associated monocyte that correlates with disease development in patients with cirrhosis. This provides a possible therapeutic target for alleviating inflammation and improving survival in cirrhosis.

**Supplementary Information:**

The online version contains supplementary material available at 10.1007/s10238-024-01305-3.

## Introduction

Cirrhosis is one of the leading causes of global mortality, resulting in over 1 million deaths annually [1]. In the progression of cirrhosis, inflammation plays a pivotal role in the progression from chronic liver disease to cirrhosis. Inflammation can be triggered by the recognition of distinctive molecules derived from pathogen production and released from dying or injured cells, known as pathogen-associated molecular patterns (PAMPs) and damage-associated molecular patterns (DAMPs), respectively [2]. Monocytes/macrophages serve as key initiators in this pathophysiological process. DAMPs and PAMPs recognize pattern recognition receptors in monocytes/macrophages, activating the NF-κb signaling pathway and leading to inflammatory cytokine production [3]. Functional activation and quantity alteration of monocytes/macrophages were reported to be closely related to cirrhosis severity from Child‒Pugh A to C and acute-on-chronic liver failure (ACLF). Monocytes enhance surface expression of HLA-DR along with costimulatory molecules CD80 and CD86, while also producing significant amounts of proinflammatory cytokines such as IL-6, TNF-α, and IL-1β [4]. The macrophage activation markers sCD206 and sCD163 show elevated levels corresponding to disease severity and exhibit good predictive value for adverse outcomes [5]. Growing evidence indicates that monocytes and macrophages are a heterogeneous population of myeloid cells [6]. In patients with chronic liver disease, a proinflammatory subset known as CD14^+^CD16^+^ monocytes, has increased both in peripheral blood and liver [7]. Additionally, an intrahepatic CD14^+^CD206^+^HLA-DR^hi^ myeloid subset was reported in pathologic liver in patients with end-stage liver disease, which spontaneously secreted proinflammatory mediators [8].

CD169, also known as sialoadhesin or Siglec-1, defines a unique macrophage subset [9]. CD169^+^ macrophages have been reported to play a critical role in various immunological regulation processes, such as antigen presentation, antitumor immunity, immunological tolerance and inflammation [10]. CD169 is constitutively expressed at low levels in monocytes. However, its expression increases dramatically in patients with an elevated IFN-α signature, due to pathogen-induced inflammation (e.g., virus infection) or nonpathogen-induced inflammation (e.g., systemic lupus erythematosus, coronary artery disease and cancer) [11–15]. Nevertheless, the precise role of CD169^+^ monocytes/macrophages has not been fully depicted in liver disease.

In this study, we observed that CD169^+^ monocytes progressively increased with the advancement of liver cirrhosis and exhibited heightened production of inflammatory mediators. Additionally, we found that CD169^+^ monocytes/macrophages displayed activated phenotypes and prominently expressed chemokine receptors. Repeated liver injury and fibrin deposition are the pathophysiological basis of cirrhosis. We showed that depletion of CD169^+^ cells in CD169-diphtheria toxin receptor (CD169-DTR) transgenic mice resulted in attenuated inflammatory response and tissue necrosis during liver injury.

## Method

### Patients and samples

Healthy controls and patients with cirrhosis in different stages were enrolled in this study. The diagnosis of cirrhosis was established based on liver biopsy, radiological evidence and/or clinical findings [16]. The patients with cirrhosis who presented one or more symptoms of ascites, upper gastrointestinal bleeding or hepatic encephalopathy (HE) were defined as decompensated. The exclusion criteria included: (1) autoimmune diseases, (2) acquired immune deficiency (human immunodeficiency virus infection), (3) immunosuppressive drug treatment, and (4) solid or hematological malignancy. The demographic and clinical information is summarized in Supplementary Table 1. Healthy controls (HCs) matched by age and sex were recruited from the Physical Examination Center at the same time.

The cirrhotic liver tissues were obtained from living donor liver transplantation (Shulan (Hangzhou) Hospital, Hangzhou, China). As a control, healthy liver tissue specimens were obtained after surgery for hemangioma. All blood samples from cirrhotic subjects were obtained from the First Affiliated Hospital, Zhejiang University School of Medicine. Written informed consent was obtained from individual subjects, and the experimental protocol was approved by the Ethics Committee of the same hospital.

### Mice

CD169-DTR mice (C57BL/6 background) were obtained from Biocytogen (Beijing, China). Mice were housed under specific pathogen-free conditions at Zhejiang University. The study conformed to the Guidelines of the China Animal Welfare Legislation and was approved by the research ethics committee of the First Affiliated Hospital of Zhejiang University School of Medicine. All mice were used for experiments at 6–8 weeks and matched for age and sex. For CD169^+^ cell depletion, DT (40 ng/g in PBS, Calbiochem) or PBS control was administered to CD169-DTR mice by caudal vein injection. Acute liver failure models were established 1 d after DT injection. For the LPS/D-Gal model, LPS (10 μg/kg, InvivoGen) and D-Gal (700 mg/kg, Sigma) were intraperitoneally (i.p.) injected. For the APAP model, mice were i.p. injected with APAP (400 mg/kg) after overnight fasting. Serum and liver tissue were collected at 1 h or 6 h after LPS/D-Gal injection, respectively, and for the APAP model, they were collected at 24 h. Hepatic fibrosis was induced by i.p. CCl_4_ (1 μl/g, Adamas) diluted 1:4 in olive oil (Sangon Biotech) twice a week for 4 weeks (nine injections).

Additional information on the materials and methods can be found in the supplementary methods and Supplementary Table 2.

## Result

### CD169^+^ monocytes in peripheral blood are correlated with inflammation and disease progression in patients with cirrhosis

**Fig. 1:**
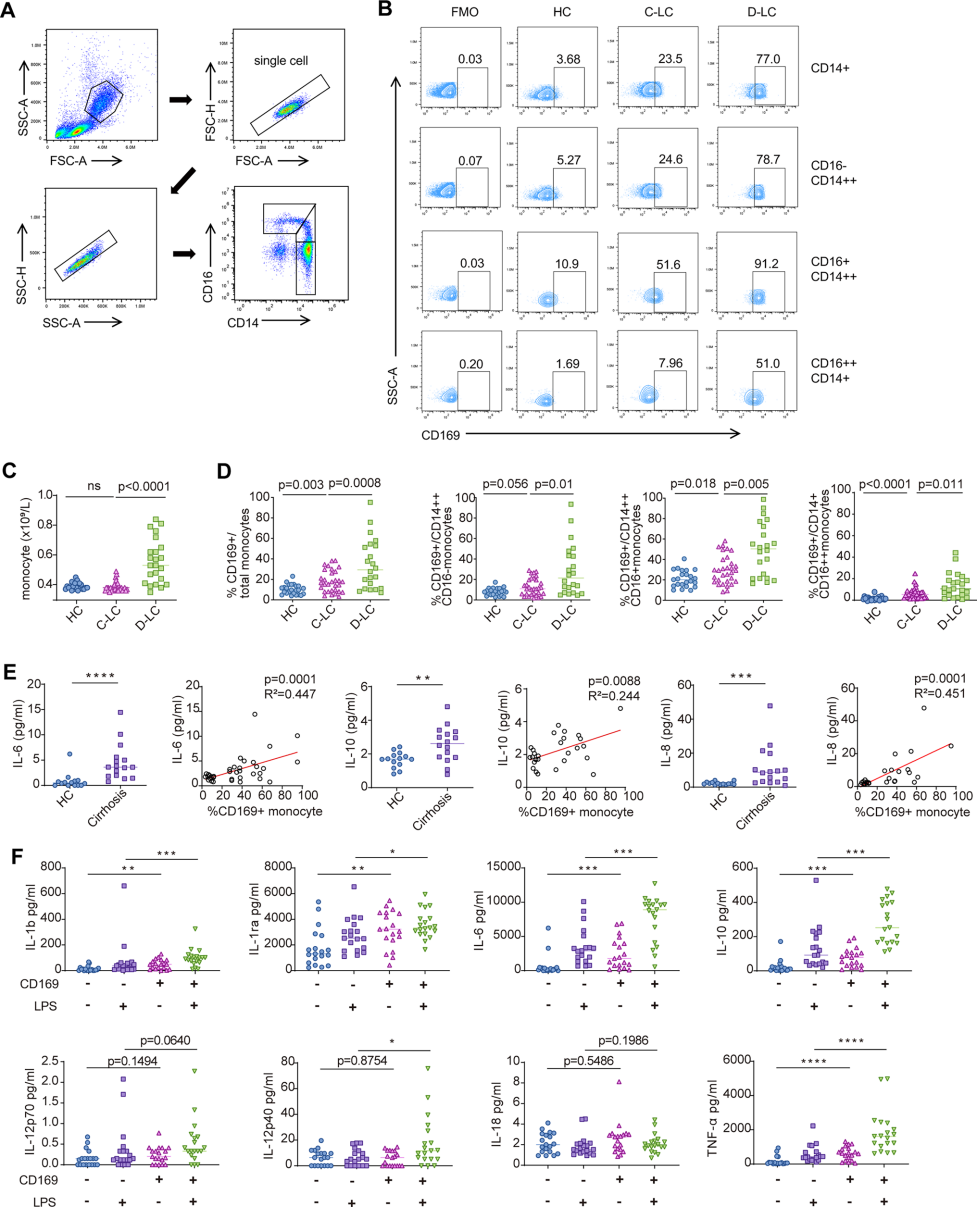
The expression of CD169 on monocytes correlates with systemic inflammation and disease severity in patients with cirrhosis. **a** Flow cytometry gating strategy of monocyte subsets. **b** CD14-positive cells were defined as monocytes, and CD16 was applied to distinct monocyte subpopulations (classical: CD14^++^CD16^−^, intermediate: CD14^++^CD16^+^ and nonclassical: CD14^+^CD16^++^). **c** The number of total monocytes in HCs and patients with different stages of cirrhosis (fibrosis, compensated cirrhosis: C-LC and decompensated cirrhosis: D-LC). **d** Expression of CD169 on monocyte subset changes between HCs and patients with different stages of cirrhosis. **e** The different plasma levels of IL-6, IL-10 and IL-8 between HCs and patients with cirrhosis and their correlation with CD169 expression on monocytes. **f** Differences in the production of inflammatory cytokines between CD169^+^ and CD169^−^ monocytes after 24 h of ex vivo culture with or without LPS stimulation

The expression of CD169 on monocytes was significantly increased in patients at the cirrhosis stage compared to healthy control (HC) (Fig. 1a-b, d). The number of monocytes was significantly increased in patients with decompensated liver cirrhosis (D-LC) (Fig. 1c). When the disease progressed from compensated liver cirrhosis(C-LC) to D-LC, CD169 was significantly elevated in all subsets along with the progression (Fig. 1d). Furthermore, we detected the differential expression of CD169 on different monocyte subpopulations (Fig. 1d). Monocytes in humans are generally divided into three subsets: classical CD14^++^CD16^−^, intermediate CD14^++^CD16^+^ and nonclassical CD14^+^CD16^++^ [7]. We found that CD169 was much more expressed in the intermediate subset. Next, we detected plasma inflammatory mediators (IL-6, IL-10, TNF-α, IL-8 and IL-12p70) in patients with cirrhosis and controls (Fig. 1e). The results indicated that IL-6, IL-10 and IL-8 were higher in cirrhosis patients than in healthy controls. The increased expression of CD169 was positively correlated with IL-6, IL-10 and IL-8 (Fig. 1e). When cultured in vitro for 24 h, the CD169^+^ subset secreted higher levels of many cytokines than the CD169^−^ subset, including IL-6, IL-1b, IL-1ra, TNF-α and IL-10, regardless of LPS stimulation (Fig. 1f). IL-12 and IL-18 levels were not significantly different between the two monocyte subsets (Fig. 1f). Thus, CD169 defines activated monocytes and is associated with high production of inflammatory cytokines.

### CD169^+^ monocytes exhibit mixed M1 and M2 phenotypes and express high levels of chemokine receptors

**Fig. 2:**
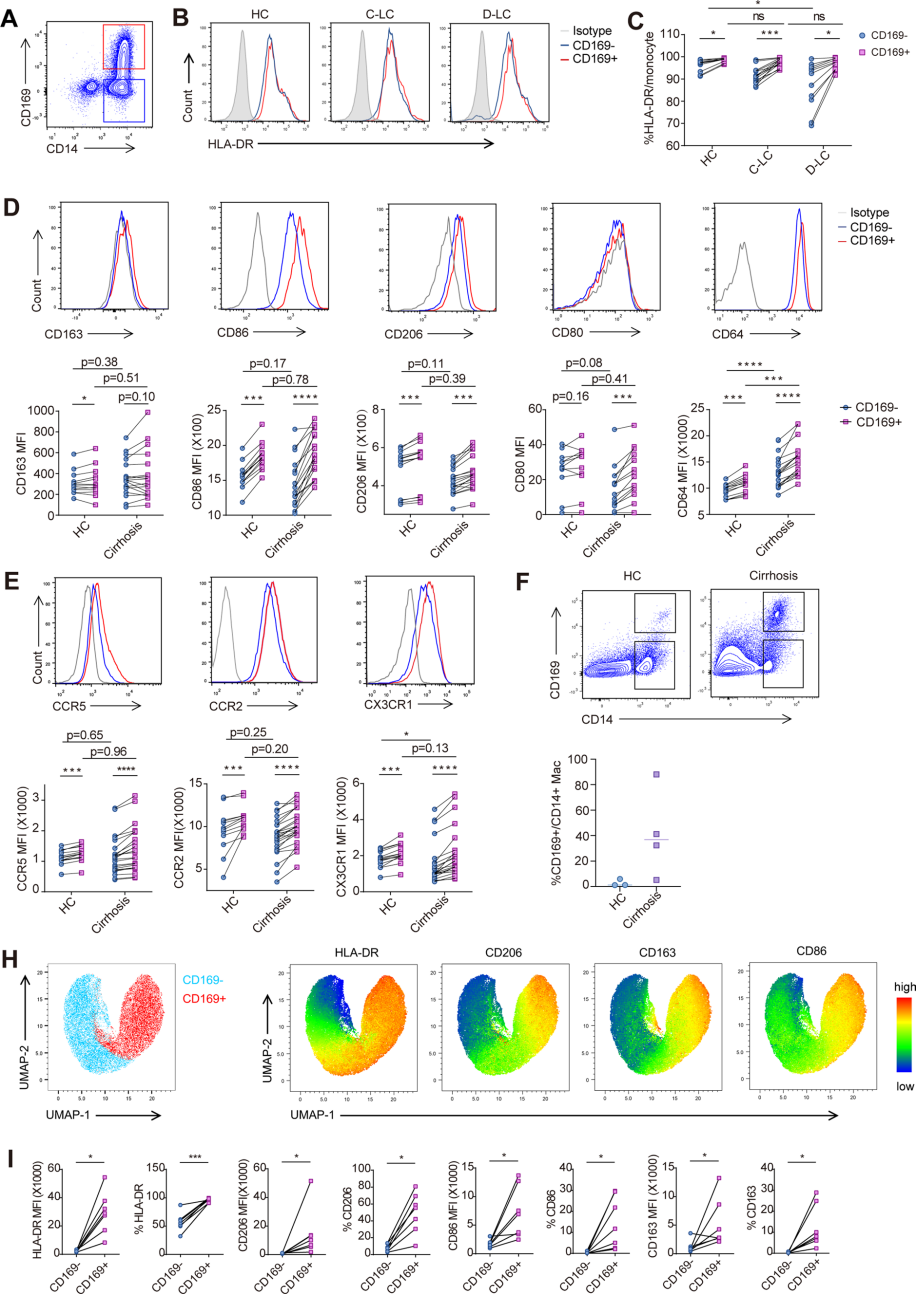
Phenotype and chemokine receptor analysis of CD169^+^ monocytes in peripheral blood and liver tissue. **a** Flow cytometry gating strategy of CD169^+^ and CD169^−^ monocyte subsets. **b**, **c** Representative charts and statistical analysis of HLA-DR expression on CD169^+^ and CD169^−^ monocytes in peripheral blood. **d** The differential expression of M1 markers (CD80, CD86 and CD64) and M2 markers (CD206 and CD163) between peripheral blood CD169^+^ and CD169^−^ monocytes in HCs and cirrhosis patients. **e** The differential expression of chemokine receptors (CCR2, CCR5 and CX3CR1) between peripheral blood CD169^+^ and CD169^−^ monocytes in HCs and cirrhotic patients. **f-g** Representative charts and statistical analysis of the percentage of CD169^+^ monocytes in normal liver and cirrhotic tissue. **h–i** The t-SNE projection and statistical analysis of polarization marker (HLA-DR, CD86, CD206, and CD163) expression on CD169^+^ and CD169^−^ monocytes in liver tissue

Furthermore, we investigated the phenotypic differences between CD169^+^ and CD169^−^ monocytes in peripheral blood and liver tissue from patients with cirrhosis. The CD169^+^ monocytes in peripheral blood exhibited both phenotypic characteristics of classically activated M1-like macrophages (HLA-DR and CD86) and an alternatively activated M2-like macrophage phenotype (CD206) (Fig. 2a–d). All these markers were expressed at higher levels on CD169^+^ monocytes than on CD169^+^ monocytes but showed no significant difference between cirrhotic patients and healthy controls. We observed no significant difference in CD163 expression between the two subsets (Fig. 2d). CD64 is an FCγ receptor of immunoglobulin G and increases surface expression on monocytes and granulocytes in conditions of infection and systemic inflammation [17]. In cirrhosis, both CD169^+^ and CD169^−^ monocytes had increased surface expression of CD64 in comparison with healthy controls, while CD169^+^ monocytes had higher expression than CD169^−^ monocytes (Fig. 2d). The expression of chemokine receptors also differed between CD169^+^ and CD169^−^ monocytes. CCR2, CCR5 and CX3CR1 were expressed at much higher levels on CD169^+^ monocytes than on CD169^−^ monocytes (Fig. 2e).

In human cirrhotic liver, we found a significant accumulation of CD14^+^CD169^+^ cells when compared with controls (Fig. 2f). Further analysis indicated that the phenotypic characteristics of CD14^+^CD169^+^ cells in the liver were similar to those in peripheral blood. HLA-DR expression was higher in the CD169^+^ subset than in the CD169^−^ subset. CD206, CD86 and CD163 were dominantly expressed in the CD169^+^ subset (Fig. 2h and i).

### CD169-expressing monocytes exhibit an immune activation profile and are induced by Toll-like receptor (TLR) activation

**Fig. 3:**
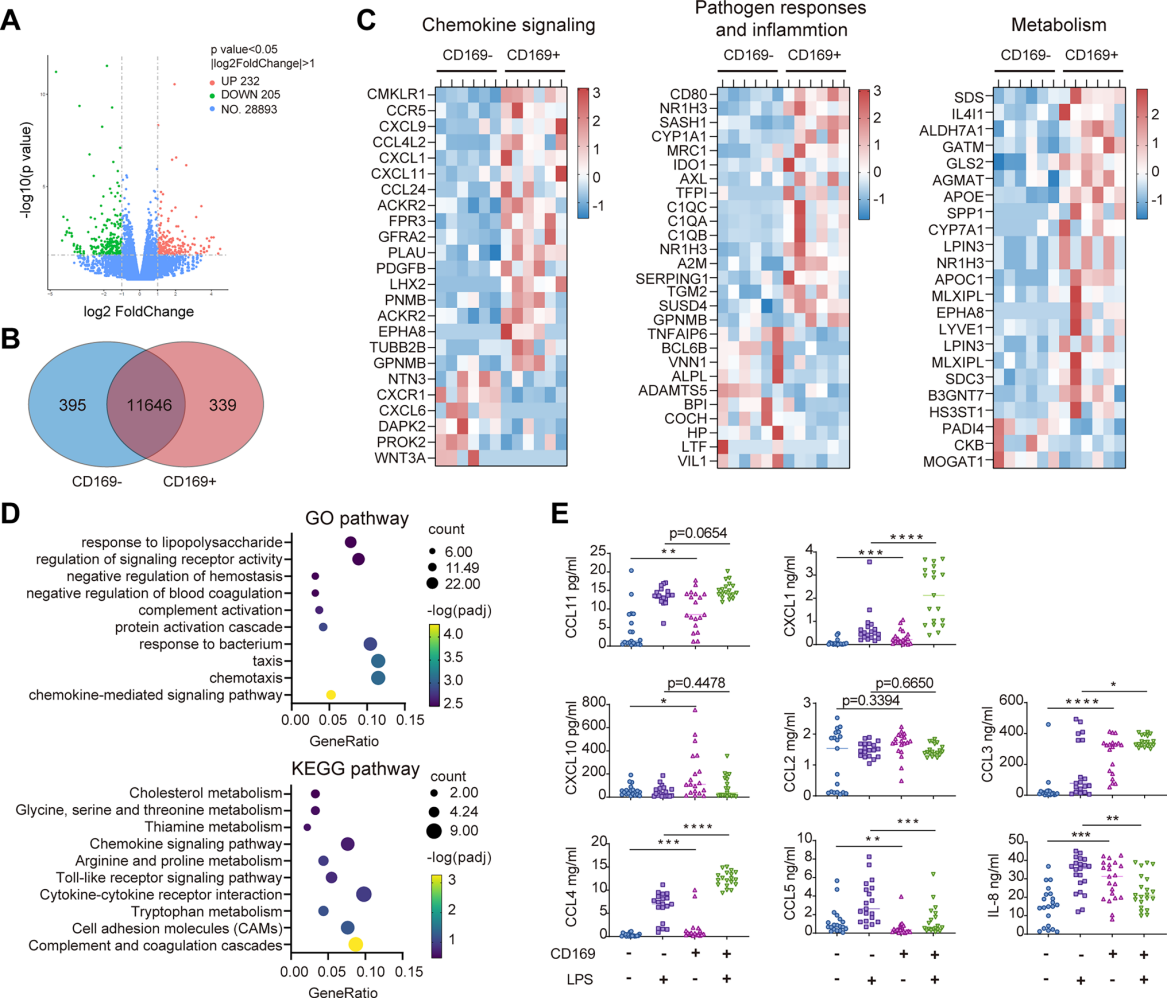
CD169^+^ monocytes displayed immune-activated profiles in patients with cirrhosis. **a-b** RNA-seq was performed to compare transcriptome differences between CD169^+^ and CD169^−^ monocytes sorted from 6 patients with cirrhosis. The significantly differentially expressed genes are displayed by volcano plots and Venn diagrams. **c** Heatmap showing significantly differentially expressed genes in various pathways. **d** KEGG and GO analysis displayed the significant ﻿signaling pathways that were enriched by differential genes. **e** Differences in the production of chemokines between CD169^+^ and CD169^−^ monocytes after 24 h of ex vivo culture with or without LPS stimulation

To fully characterize CD169^+^ monocytes in cirrhosis, CD169^+^ and CD169^−^ monocytes from 6 patients with cirrhosis were sorted and subjected to transcriptome analysis. The volcano plot indicated that 232 genes were upregulated, and 205 genes were downregulated in CD169^+^ monocytes (p < 0.05) (Fig. 3a). The Venn diagram showed that 399 genes were specifically expressed in CD169^+^ monocytes, while 395 genes were specifically expressed in CD169^−^ monocytes (Fig. 3b). The Gene Ontology (GO) analysis to assess the involved biological process revealed that the differential genes between CD169^+^ and CD169^−^ monocytes were predominantly related to chemokine or chemotaxis and pathogen responses (Fig. 3d). The KEGG pathway analysis also indicated that the chemokine signaling pathway and Toll-like receptor pathway were significantly different between the two monocyte subsets (Fig. 3d). The different subsets exhibited different chemokine expression, which indicated upregulation of CXCL9, CCL4L2, CXCL1 and CXCL11 but downregulation of CXCL6 in CD169^+^ monocytes (Fig. 3c). To further clarify the differential expression of chemokines, we compared the levels of chemokines in the culture supernatant of CD169^+^ and CD169^−^ monocytes with or without LPS stimulation for 24 h. We found that without LPS stimulation, CCL11, CXCL1, CXCL10, CCL3, CCL4 and IL-8 were secreted at significantly higher levels in CD169^+^ monocytes than CD169^−^ monocytes (Fig. 3e). In contrast, CCL5 was reduced in CD169^+^ monocytes (Fig. 3e). Under LPS stimulation, the expression of CXCL1, CCL3 and CCL4 remained higher in CD169^+^ monocytes than in CD169^−^ monocytes (Fig. 3e). The heatmap of inflammatory genes revealed that CD169^+^ monocytes expressed complex activated markers that upregulated polarization molecules for both M1 (CD80, IDO1 and CXCL9) and M2 (MRC1, TGM2, AXL, C1QA/B/C) (Fig. 3c). A variety of metabolism-related genes was upregulated in CD169^+^ monocytes, which also illustrated the immune-activated phenotype, including cholesterol metabolism (APOE, APOC1 and CYP7A1) and arginine and proline metabolism (ALDH7A1, GATM, CKB and AGMAT) (Fig. 3c).

**Fig. 4:**
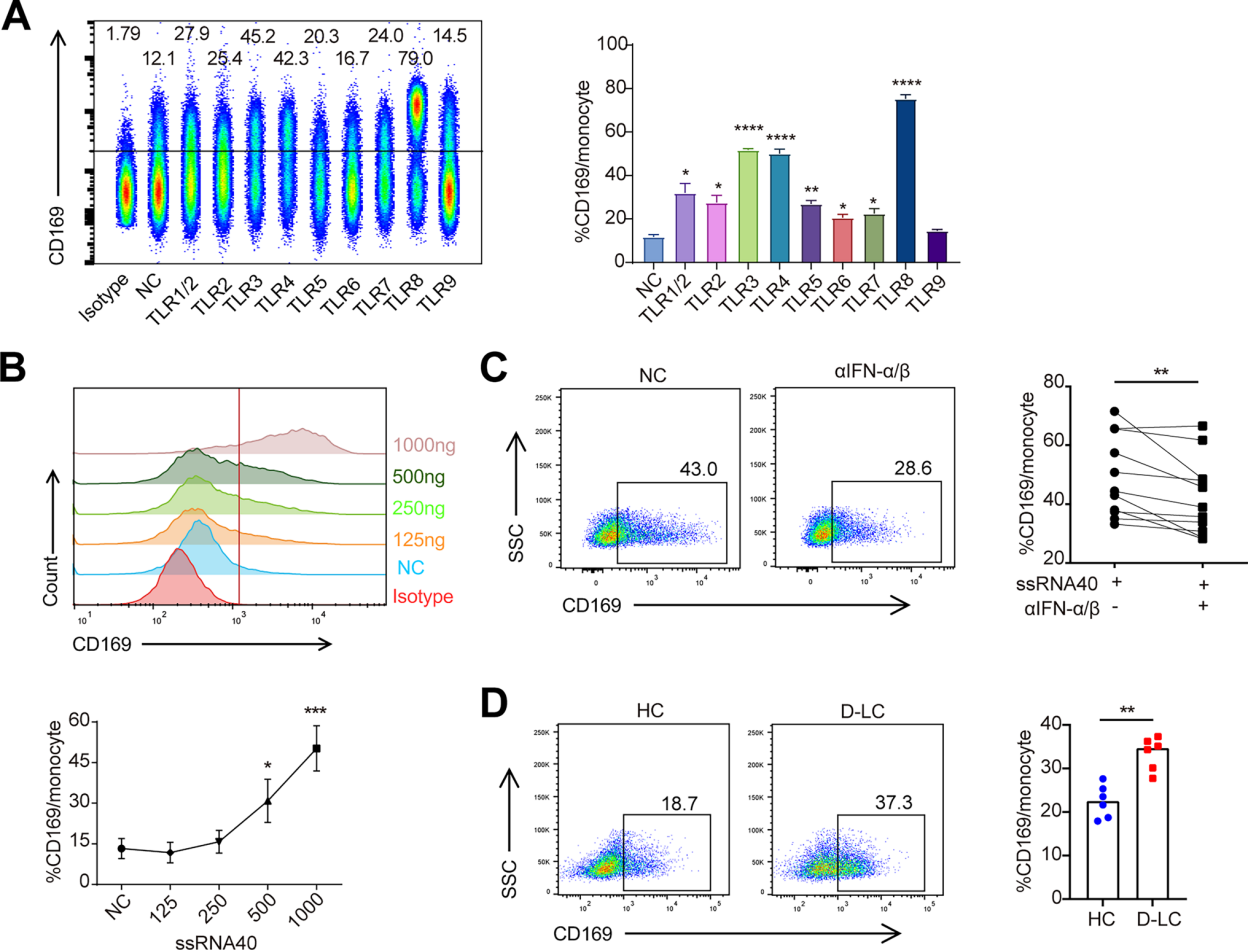
TLR agonists induce expression of CD169 in monocytes. **a** CD169 expression on the surface of monocytes after stimulation with TLR agonists. **b** CD169 expression on the surface of monocytes after stimulation with a TLR8 agonist (ssRNA40) at different concentrations. **c** CD169 expression on the surface of monocytes under stimulation with a TLR8 agonist after neutralizing IFN-α/β. **d** upregulating CD169 expression on the surface of monocytes upon stimulation with plasma from D-LC

Bacterial products or dying cell components trigger the activation of pattern recognition receptors (PRRs) and result in systemic inflammation. To assess the effect of TLR1-9 stimulation on CD169 expression, the surface expression of CD169 on monocytes was detected by flow cytometry. Stimulation with TLR agonists upregulated CD169 expression on healthy monocytes (Fig. 4a). Among those agonists, the TLR8 agonist (ssRNA40) is the most robust agonist in the induction of CD169 (Fig. 4b). The activated TLR leads to the production of type I IFN. Blocking IFNα/β significantly attenuated the expression of CD169 after TLR8 stimulation (Fig. 4c). We also devised a disease-mimicking model whereby freshly isolated CD14^+^ monocytes from healthy doner were incubated with plasma either from D-LC or HC. The result indicated plasma from D-LC effectively upregulated expression of CD169 on monocytes (Fig. 4d).

### CD169^+^ monocytes promote neutrophil survival through G-CSF and GM-CSF

**Fig. 5:**
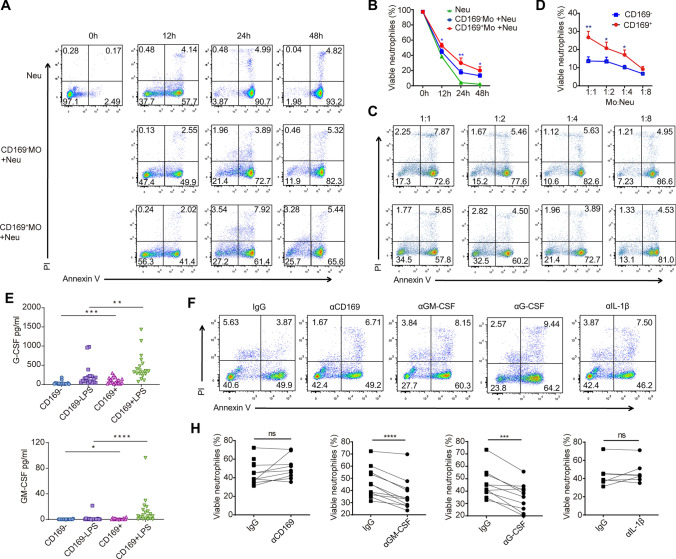
CD169^+^ monocytes robustly promote neutrophil survival via high G-CSF and GM-CSF expression. **a**, **b** Representative charts and statistical analysis of neutrophil apoptosis and survival after coculture of CD169^+^ or CD169^−^ monocytes with neutrophils at 12 h, 24 h and 48 h. **c**, **d** Representative charts and statistical analysis of neutrophil apoptosis and survival after coculture of CD169^+^ or CD169^−^ monocytes with neutrophils at different ratios. **e** The concentrations of GM-CSF and M-CSF in the culture supernatant of CD169^+^ or CD169^−^ monocytes with or without LPS stimulation. **f** Representative charts and statistical analysis of neutrophil apoptosis and survival after blocking CD169, GM-CSF, G-CSF or IL-1β in the neutrophil and CD169^+^ monocyte coculture system

Neutrophils are recruited to infection or injury sites as first-action leukocytes, where they eliminate pathogens through phagocytosis and release proteolytic enzymes, antimicrobial proteins, and reactive oxygen species [18]. These released substances are responsible for local inflammation and induce host tissue damage in a variety of pathological conditions. Therefore, to minimize host injury, it is critical to rapidly eliminate neutrophils via cell death. We performed a series of experiments to determine whether CD169^+^ monocytes were more effective in keeping neutrophils alive than CD169^−^ monocytes in vitro. CD169^+^ monocytes and CD169^−^ monocytes were sorted from the peripheral blood of cirrhotic patients. Then, neutrophils from healthy controls were cocultured with different monocytes. We confirmed that CD169^+^ monocytes can also modulate neutrophil survival (Fig. 5a–d). We found that the percentage of neutrophil apoptosis increased with culture time in vitro, but coculturing with monocytes effectively diminished neutrophil apoptosis. Although coculture with both CD169^+^ monocytes and CD169^−^ monocytes inhibited neutrophil apoptosis, CD169^+^ monocytes were much more robust than CD169^−^ monocytes. We found that the higher the monocyte ratio was, the lower the neutrophil apoptosis after 24 h. Next, we tested whether the antiapoptotic function of CD169^+^ monocytes occurred via CD169-mediated cell‒cell contact via CD169. As a result, we found that neutrophil apoptosis was not significantly increased after blocking CD169 in the coculture system (Fig. 5f and h). The soluble mediators secreted by CD169^+^ monocytes may contribute to neutrophil survival. Previous evidence has indicated that GM-CSF and G-CSF are robust antiapoptotic cytokines that upregulate Survivin [19]. Additionally, IL-1β also regulates neutrophil apoptosis [20, 21]. We found that CD169^+^ monocytes expressed higher levels of GM-CSF and G-CSF than CD169^−^ monocytes, and similar results were observed after LPS stimulation (Fig. 5e). Neutralizing GM-CSF and G-CSF but not IL-1β attenuated the antiapoptotic effect after coculture with CD169^+^ monocytes and neutrophils (Fig. 5f and h).

### Specific depletion of CD169^+^ cells attenuates liver injury but impairs fibrosis resolution in mouse model

**Fig. 6:**
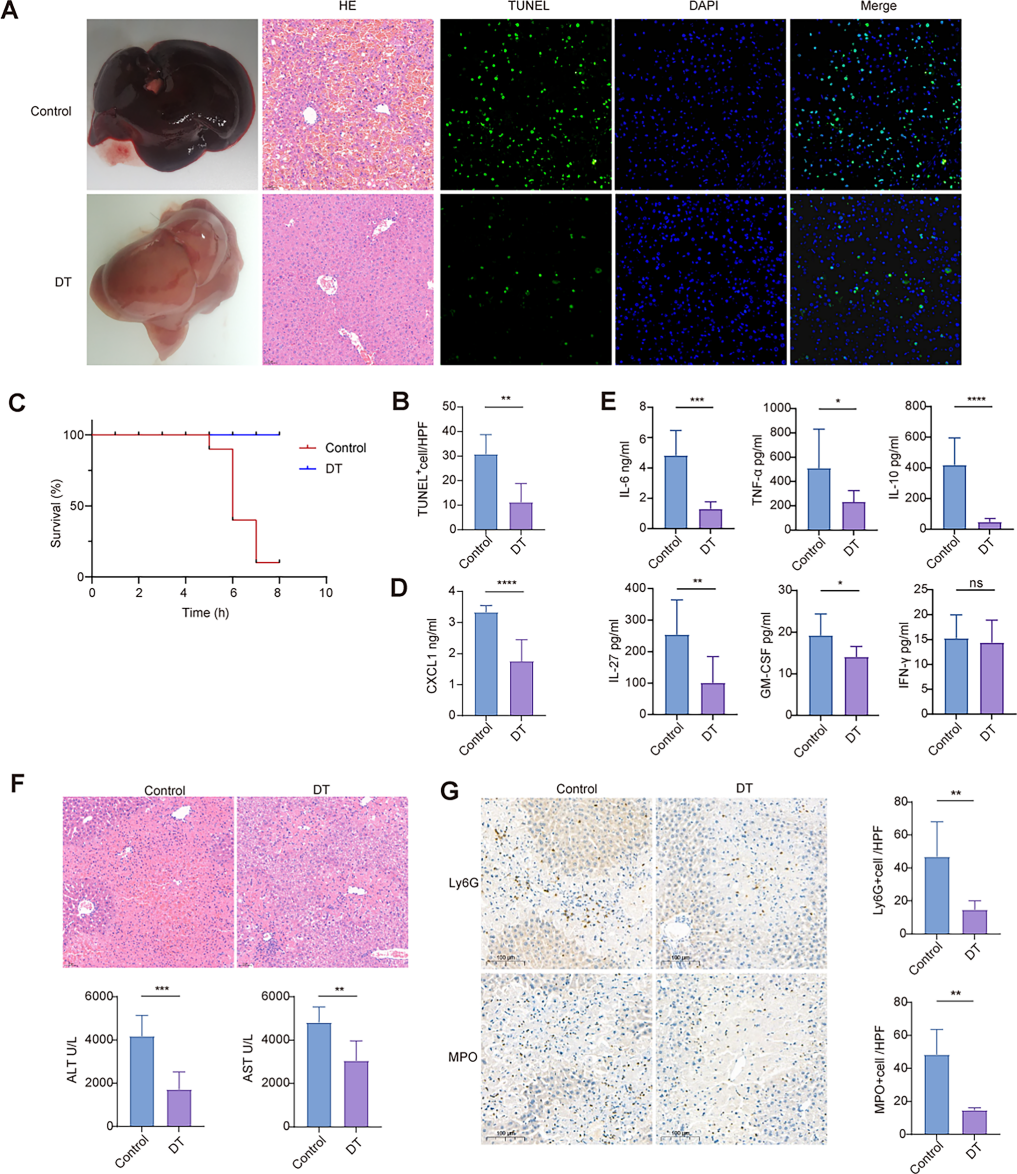
Depleting CD169^+^ cells alleviates acute liver injury in a mouse model. **a**, **b** Depletion of CD169^+^ cells using diphtheria toxin significantly alleviated liver necrosis in LPS/D-GalN-induced liver injury. **c** The survival curve indicated that CD169-DTR mice had an increased survival rate. **d**, **e** Inflammatory mediators (CXCL1, IL-6, TNF-α, IL-27, IL-10, GM-CSF and IFN-γ) were measured in plasma. **f** HE staining and plasma levels of ALT and AST showed that liver necrosis was significantly alleviated in CD169-depleted mice in comparison with WT mice in APAP-induced liver injury. **g** Ly6G and MPO indicated that neutrophil infiltration was decreased in D169-depleted mice

Repeated liver injury and fibrin deposition are the key pathophysiological mechanisms of cirrhosis. We sought to understand the influence of cirrhotic progression in the absence of CD169^+^ cells by using CD169-DTR transgenic mice. In the liver, the different macrophage subpopulations showed different surface expression of CD169 (Figure S1A). The two KC (Kupffer cell) subpopulations (KC1 CD206^−^ESAM^−^ and KC2 CD206^+^ESAM^+^) highly expressed CD169 and showed no difference between the two subsets. The capsular macrophage subset (TIM4^−^I-A/I-E^+^) displayed lower CD169 expression than KCs but higher CD169 expression than monocyte-derived macrophages (MoMF, CD11b^hi^F4/80^int^). Depletion of CD169^+^ macrophages upon the administration of diphtheria toxin (DT), we observed that the KC subset was entirely eliminated, and the ratio of Ly6C^hi^/Ly6C^lo^ subsets in MoMF was also dramatically changed (Figure S1B). KCs exert an anti-inflammatory role in acute liver injury. Loss of KCs and infiltration of monocytes promote an inflammatory environment in acute liver insult [22]. We next utilized two established models of liver injury to understand the physiological role of CD169^+^ macrophages during acute liver injury. Interestingly, we found that compared with WT mice, CD169-DTR mice exhibited less severe liver injury in pathology and lower mortality after administration of LPS and D-GalN (Fig. 6a and b). The TUNEL assay is a well-established method to detect cell death-associated DNA fragmentation. The results also showed that CD169-depleted mice presented fewer TUNEL-positive cells in the liver (Fig. 6c). CD169-depleted mice also had significantly decreased plasma levels of inflammatory mediators, including IL-6, TNF-α, IL-10, GM-CSF and CXCL1 (Fig. 6d and e). The rodent model of APAP-induced liver injury also reached the same conclusion. In the absence of CD169^+^ cells, APAP-induced acute liver injury was alleviated, which represented a lower severity of liver necrosis and low levels of plasma AST and ALT (Fig. 6f). In addition, eliminating CD169^+^ cells decreased neutrophil infiltration in the liver, which may also contribute to diminishing acute liver injury after APAP injection (Fig. 6g).

**Fig. 7:**
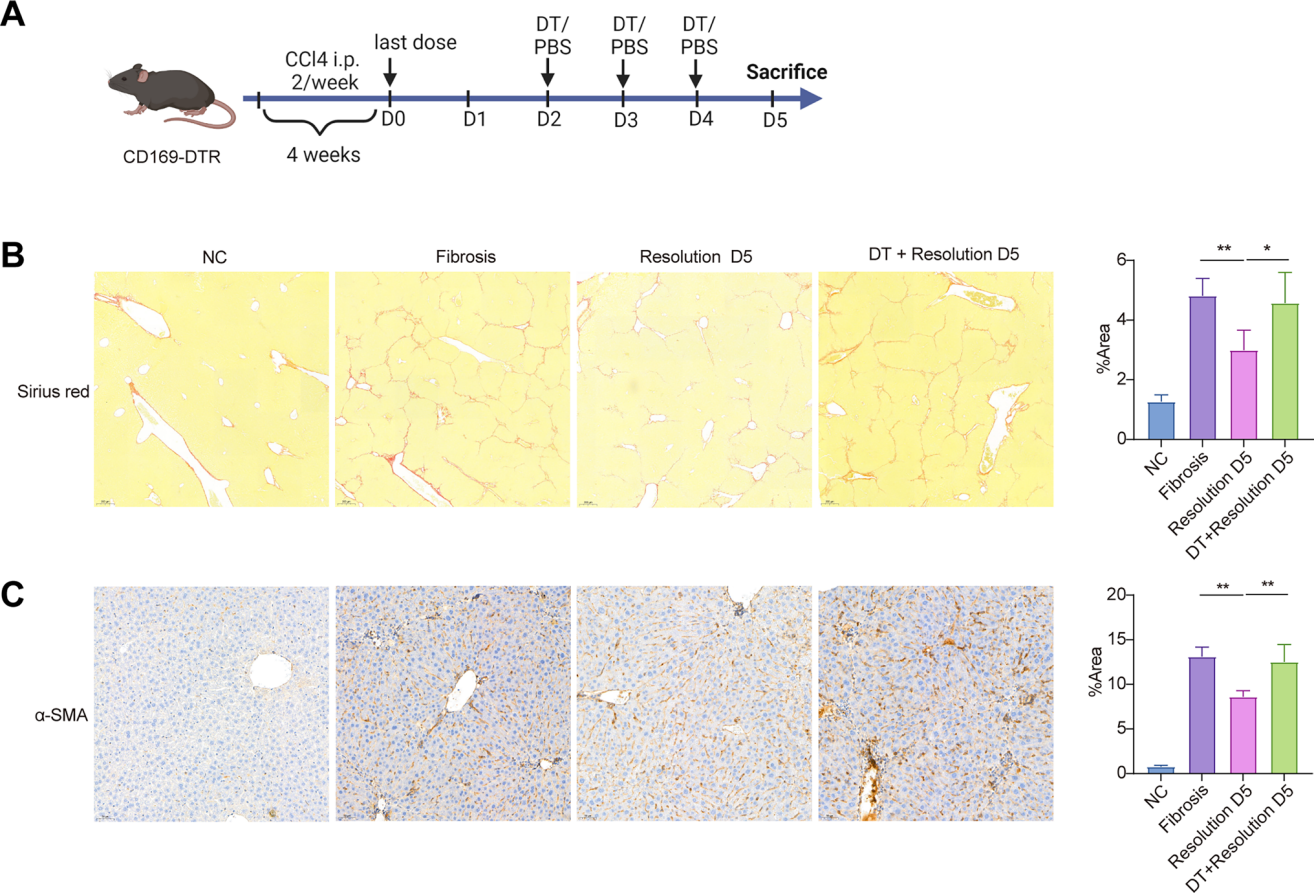
Depleting CD169^+^cells impair spontaneous scar resolution in mice model. **a** the Schematic diagram of spontaneous scar resolution mice model. **b**, **c** The Sirius red and α-SMA indicated the fibrin deposition and activation of hepatic stellate cell, respectively, in CD169-depleting and WT mice

To define the functional role of distinct CD169^+^ subset in mediating scar resolution, the CD169-DTR mice were given CCl_4_ for 4 weeks and then, the spontaneous scar resolution was observed. After last dose of CCl_4_, the mice were administrated DT from day 2 to day 5 for inducing significant depletion of the target subset until harvest (Fig. 7a). The CD169-depleted mice directly impaired spontaneous resolution of liver fibrosis (Fig. 7b). The differences were also detected in the α-SMA area after CD169^+^ subset depletion, suggesting that the observed phenotype may be related to continuing myofibroblast activation (Fig. 7c). In order to determine effect of DT treatment itself on fibrosis resolution, we examined administration of DT or PBS in WT mice during the spontaneous resolution model. The results indicated that DT administration has no effect on scar resolution in WT mice (Figure S2).

## Discussion

In this study, we confirmed an increase in the CD169^+^ monocyte subset with the severity of cirrhosis, which robustly contributes to systemic and local inflammation. This was characterized by elevated expression of the inflammatory cytokines IL-6, IL-1β, TNF-α and IL-10, as well as enhanced neutrophil recruitment and survival. Depletion of CD169^+^ cells in CD169-DTR transgenic mice effectively attenuated the inflammatory response and hepatic necrosis in an acute liver injury model.

Systemic inflammation plays a critical role in the progression of cirrhosis [23]. Previous studies have shown that cirrhotic patients with bacterial infection exhibit excessive production of the inflammatory cytokines TNF-α and IL-6, which is associated with the development of an episode of extrahepatic organ failure and in-hospital mortality [24]. Studies which stimulated monocytes or PBMCs from patients with cirrhosis or healthy controls with LPS ex vivo have indicated higher level of TNF-α, IL-1β and IL-6 production in the former [25, 26]. Despite extensive research, the mechanism underlying the excessive proinflammatory response to LPS in patients with cirrhosis remains largely unclear. The gram-negative bacterial component LPS can induce “antiviral” IFN-stimulated gene (ISG) expression even without a viral pathogen. Evidence indicates that during the progression of liver fibrosis, the gut microbiota and bacterial degradation products are delivered via portal venous blood to the liver and circulation, triggering tonic type I IFN expression, which results in dysfunction of innate immune responses and an inability to control bacterial infection [27]. Increased activation of the IFN signaling pathway can result in excessive inflammation and tissue damage. In patients with alcoholic cirrhosis, higher baseline expression of ISGs was associated with a higher risk of death [28]. Expression of CD169 on circulating monocytes as an indicator of type I IFN activation [13]. A high level of IFN-α in systemic lupus erythematosus (SLE) patients demonstrated high expression of CD169 on monocytes and contributed to disease activation [29]. Furthermore, exposure of healthy monocytes to IFN-α robustly induces CD169 expression [30]. Our findings confirmed that the CD169-expressing monocyte population expanded with the progression of liver cirrhosis. The high level of CD169 expression may indicate the activation of the IFN signaling pathway. It is worth noting that CD169^+^ monocytes from patients with cirrhosis highly express both IL-10 and IL-6. On the one hand, IL-6 is a sensitive marker of systemic inflammation and is closely related to mortality [23, 31]. On the other hand, IL-10, a key immunosuppressive mediator, causes corruption of antibacterial immunity, leading to loss of infection control and infection-associated mortality [27]. Therefore, increased levels of both pro- and anti-inflammatory cytokines may help to explain the immunodeficiency and systemic inflammation that are present in cirrhosis. Interestingly, we also found that the intrahepatic and circulating CD169^+^ CD14^+^ myeloid cells in cirrhotic patients displayed “mixed” phenotypes, highly expressing both M1 and M2 macrophage markers. Considering the high degree of plasticity of macrophages, the simple M1/M2 dichotomy cannot properly reflect the complex phenotypic changes in cirrhosis. It is not uncommon to find macrophages with both pro- and anti-inflammatory phenotypes simultaneously in the same tissue [32]. A chronic alcohol feeding mouse model indicated an increased frequency of CD206^+^CD163^+^ macrophages and led to increased expression of M1 (TNF-α, MCP1, and IL-1β) and M2 (Arg1, Mrc1, and IL-10) genes [33]. In both a mouse model of HBV-induced liver inflammation and patients with advanced viral-related chronic liver disease, the intrahepatic CD14^+^HLA-DR^high^CD206^+^ myeloid population is proinflammatory and spontaneously produces high levels of TNF-α [8]. The antibiotic-mediated deletion of the intestinal microbiota caused a pronounced reduction in these cells in the liver [8]. The accumulation of CD14^+^HLA-DR^high^CD206^+^ in pathological liver is directly linked to gut intestinal microbiota but not viral infection. Our data supported that the CD169^+^CD14^+^ subset may share the same phenotypes and functions as the intrahepatic myeloid subset reported by Alfonso Tan-Garcia and colleagues [8]. Elevated CD169^+^CD14^+^ myeloid cells help to further explain that the mechanisms of type I IFN are critical in inducing systemic inflammation and immune deficiency in patients with cirrhosis. However, how the activation of the IFN signaling pathway by bacterial products shifts monocytes/macrophages toward “mixed” features in this course needs further study.

Neutrophils are the largest population of circulating myeloid cells and a key component of local inflammation. During local inflammation, neutrophils are recruited from the circulation as first-action leucocytes and perform a series of antibacterial functions, including degranulation, ROS generation, phagocytosis, and the formation of neutrophil extracellular traps (NETs) [34]. However, massive accumulation of neutrophils aggravates liver inflammation and tissue damage by increasing ROS, proteases, and inflammatory mediators [35]. Ex vivo culture with or without LPS stimulation also suggests different chemokine expressions between CD169^+^ and CD169^−^ monocytes. Among these differences, CXCL1 and IL-8, the major neutrophil-recruiting chemokines, were secreted at higher levels in CD169^+^ monocytes than in CD169^−^ monocytes, which may attract massive infiltration of neutrophils in the inflammatory liver. Binxia Chang and colleagues proved that overproduced CXCL1 exacerbated steatohepatitis in HFD-fed mice, whereas reducing the level of CXCL1 relieved HFD plus ethanol-induced hepatic neutrophil infiltration and injury [36]. In patients with HBV-ACLF, serum CXCL1 was positively correlated with organ failure and can be a predictive marker of short-term mortality [37]. In addition, our data also revealed that CD169^+^ monocytes prolonged neutrophil survival in vitro via high expression of G-CSF and GM-CSF. Diminished neutrophil apoptosis could augment the respiratory burst and increase inflammatory cytokine production, further worsening tissue damage and sustaining chronic inflammation. ﻿The production of GM-CSF by CD169^+^ monocytes can in turn prevent LPS tolerance and maintain TNF-α during a state of chronic inflammation [8].

Liver inflammation and repeated liver injury lead to fibrosis, cirrhosis and the development of hepatocellular carcinoma. Upon removal of the etiological source of chronic injury, hepatic fibrosis can be reversed in patients and experimental rodent models [38]. CD169-DTR transgenic mice were used to explore the roles of CD169^+^ cells in immune responses to liver injury. Depleting CD169^+^ cells dramatically attenuated liver injury and the local inflammatory response in hepatitis models. The infiltration of MoMFs is a main change for augmenting liver inflammation, while loss of KCs is responsible for reducing immune tolerance [39–41]. Our results may help to illustrate that CD169^+^ MoMFs are a key proinflammatory subset of liver-infiltrated MoMFs. In addition, depleting CD169^+^ cells attenuated neutrophil recruitment in the necrotic area of the liver. Neutrophils are recruited to sites of inflammation or injured tissue as first-action myeloid cells, where they cause tissue damage though cytokine and granule protein release. Reducing neutrophil infiltration helps to ameliorate acute liver injury [42, 43].

Our findings also strengthen that pre-existed inflammation may service an indispensable role in the process of fibrosis resolution. After depleting of CD169, the spontaneous resolution of liver fibrosis was also impaired. Neutrophils are key proinflammation subset in acutes injury phase but also facilitates liver scar degradation in resolution phase after liver injury [44]. The diminished recruitment of neutrophils after CD169 cells depletion may attenuate the fibric degradation. Besides, the restorative monocyte/macrophage subset may derive from the proinflammatory monocyte/macrophage subset. Previous observations indicated in liver fibrosis resolution, the proinflammatory Ly6C^hi^ monocytes converted to restorative Ly6Clo monocytes, which exhibited highly expression of matrix-degrading metalloproteinase enzymes and enhanced phagocytosis [45]. Interestingly, we found the mice with CD169 cells depletion were prone to die by DT treatment during the fibrosis formation phase of CCl_4_ administration (Data not shown). The possible mechanisms are deserved to be explored further. The phenomenon may reveal depleting CD169-positive cells affect the hepatic drug metabolism upon CCl_4_ administration, amplifying radical peroxidation of hepatocyte lipids.

In conclusion, we identified the expansion of CD169^+^ myeloid cells along with the progression of cirrhosis in the blood and liver. The special subset expressed both M1- and M2-like macrophage phenotypes and was critical in promoting systemic inflammation in patients with cirrhosis with high expression of inflammatory cytokines and chemokines. Special depletion of CD169^+^ cells in a mouse model dramatically ameliorated acute liver injury, but impaired fibrosis resolution.

## Supplementary Information

Below is the link to the electronic supplementary material.

Supplementary file 1 (TIF 1458 KB)

Supplementary file 2 (TIF 1598 KB)

Supplementary file 3 (DOCX 18 KB)

Supplementary file 4 (DOC 43 KB)

## Data Availability

The data that support the findings of this study are available from the corresponding author upon reasonable request.

## References

[1] Duncan BB, Schmidt MI, Global Burden of Disease 2017 Cirrhosis Collaborators. The global, regional, and national burden of cirrhosis by cause in 195 countries and territories, 1990–2017: a systematic analysis for the Global Burden of Disease Study 2017. Lancet Gastroenterol Hepatol. 2020;5(3):245–66. 31981519 10.1016/S2468-1253(19)30349-8 PMC7026710

[2] Bernsmeier C, van der Merwe S, Périanin A. Innate immune cells in cirrhosis. J Hepatol. 2020;73(1):186–201. 32240716 10.1016/j.jhep.2020.03.027

[3] Albillos A, Martin-Mateos R, Van der Merwe S, Wiest R, Jalan R, Álvarez-Mon M. Cirrhosis-associated immune dysfunction. Nat Rev Gastroenterol Hepatol. 2022;19(2):112–34. 34703031 10.1038/s41575-021-00520-7

[4] Albillos A, Lario M, Álvarez-Mon M. Cirrhosis-associated immune dysfunction: distinctive features and clinical relevance. J Hepatol. 2014;61(6):1385–96. 25135860 10.1016/j.jhep.2014.08.010

[5] Grønbæk H, Rødgaard-Hansen S, Aagaard NK, et al. Macrophage activation markers predict mortality in patients with liver cirrhosis without or with acute-on-chronic liver failure (ACLF). J Hepatol. 2016;64(4):813–22. 26639396 10.1016/j.jhep.2015.11.021

[6] Tacke F, Zimmermann HW. Macrophage heterogeneity in liver injury and fibrosis. J Hepatol. 2014;60(5):1090–6. 24412603 10.1016/j.jhep.2013.12.025

[7] Liaskou E, Zimmermann HW, Li KK, et al. Monocyte subsets in human liver disease show distinct phenotypic and functional characteristics. Hepatology. 2013;57(1):385–98. 22911542 10.1002/hep.26016 PMC4194426

[8] Tan-Garcia A, Wai LE, Zheng D, et al. Intrahepatic CD206(+) macrophages contribute to inflammation in advanced viral-related liver disease. J Hepatol. 2017;67(3):490–500. 28483682 10.1016/j.jhep.2017.04.023

[9] Chávez-Galán L, Olleros ML, Vesin D, Garcia I. Much More than M1 and M2 Macrophages, There are also CD169(+) and TCR(+) Macrophages. Front Immunol. 2015;6:263. 26074923 10.3389/fimmu.2015.00263 PMC4443739

[10] Liu Y, Xia Y, Qiu CH. Functions of CD169 positive macrophages in human diseases (Review). Biomed Rep. 2021;14(2):26. 33408860 10.3892/br.2020.1402 PMC7780751

[11] Bedin AS, Makinson A, Picot MC, et al. Monocyte CD169 expression as a biomarker in the early diagnosis of coronavirus disease 2019. J Infect Dis. 2021;223(4):562–7. 33206973 10.1093/infdis/jiaa724 PMC7717347

[12] Biesen R, Demir C, Barkhudarova F, et al. Sialic acid-binding Ig-like lectin 1 expression in inflammatory and resident monocytes is a potential biomarker for monitoring disease activity and success of therapy in systemic lupus erythematosus. Arthritis Rheum. 2008;58(4):1136–45. 18383365 10.1002/art.23404

[13] York MR, Nagai T, Mangini AJ, Lemaire R, van Seventer JM, Lafyatis R. A macrophage marker, Siglec-1, is increased on circulating monocytes in patients with systemic sclerosis and induced by type I interferons and toll-like receptor agonists. Arthritis Rheum. 2007;56(3):1010–20. 17328080 10.1002/art.22382

[14] Li C, Luo X, Lin Y, et al. A higher frequency of CD14+ CD169+ monocytes/macrophages in patients with colorectal cancer. PLoS ONE. 2015;10(10): e0141817. 26509874 10.1371/journal.pone.0141817 PMC4625021

[15] Prenzler S, Rudrawar S, Waespy M, Kelm S, Anoopkumar-Dukie S, Haselhorst T. The role of sialic acid-binding immunoglobulin-like-lectin-1 (siglec-1) in immunology and infectious disease. Int Rev Immunol. 2023;42:113–38. 34494938 10.1080/08830185.2021.1931171

[16] Mokdad AA, Lopez AD, Shahraz S, et al. Liver cirrhosis mortality in 187 countries between 1980 and 2010: a systematic analysis. BMC Med. 2014;12:145. 25242656 10.1186/s12916-014-0145-y PMC4169640

[17] García-Salido A, Serrano-González A, Casado-Flores J, et al. CD64 on monocytes and granulocytes in severe acute bronchiolitis: Pilot study on its usefulness as a bacterial infection biomarker. J Leukoc Biol. 2018;103(5):965–71. 29485692 10.1002/JLB.4AB0417-152RRR

[18] Kolaczkowska E, Kubes P. Neutrophil recruitment and function in health and inflammation. Nat Rev Immunol. 2013;13(3):159–75. 23435331 10.1038/nri3399

[19] Altznauer F, Martinelli S, Yousefi S, et al. Inflammation-associated cell cycle-independent block of apoptosis by survivin in terminally differentiated neutrophils. J Exp Med. 2004;199(10):1343–54. 15148334 10.1084/jem.20032033 PMC2211817

[20] Cowburn AS, Deighton J, Walmsley SR, Chilvers ER. The survival effect of TNF-alpha in human neutrophils is mediated via NF-kappa B-dependent IL-8 release. Eur J Immunol. 2004;34(6):1733–43. 15162444 10.1002/eji.200425091

[21] Prince LR, Allen L, Jones EC, et al. The role of interleukin-1beta in direct and toll-like receptor 4-mediated neutrophil activation and survival. Am J Pathol. 2004;165(5):1819–26. 15509550 10.1016/s0002-9440(10)63437-2 PMC1618681

[22] Arroyo V, Moreau R, Jalan R. Acute-on-chronic liver failure. N Engl J Med. 2020;382(22):2137–45. 32459924 10.1056/NEJMra1914900

[23] Arroyo V, Angeli P, Moreau R, et al. The systemic inflammation hypothesis: towards a new paradigm of acute decompensation and multiorgan failure in cirrhosis. J Hepatol. 2021;74(3):670–85. 33301825 10.1016/j.jhep.2020.11.048

[24] Byl B, Roucloux I, Crusiaux A, Dupont E, Devière J. Tumor necrosis factor alpha and interleukin 6 plasma levels in infected cirrhotic patients. Gastroenterology. 1993;104(5):1492–7. 8482461 10.1016/0016-5085(93)90361-f

[25] Devière J, Content J, Denys C, et al. Excessive in vitro bacterial lipopolysaccharide-induced production of monokines in cirrhosis. Hepatology. 1990;11(4):628–34. 2184115 10.1002/hep.1840110416

[26] Tazi KA, Quioc JJ, Saada V, Bezeaud A, Lebrec D, Moreau R. Upregulation of TNF-alpha production signaling pathways in monocytes from patients with advanced cirrhosis: possible role of Akt and IRAK-M. J Hepatol. 2006;45(2):280–9. 16635535 10.1016/j.jhep.2006.02.013

[27] Hackstein CP, Assmus LM, Welz M, et al. Gut microbial translocation corrupts myeloid cell function to control bacterial infection during liver cirrhosis. Gut. 2017;66(3):507–18. 27432540 10.1136/gutjnl-2015-311224

[28] Weiss E, Rautou PE, Fasseu M, et al. Type I interferon signaling in systemic immune cells from patients with alcoholic cirrhosis and its association with outcome. J Hepatol. 2017;66(5):930–41. 28040548 10.1016/j.jhep.2016.12.008

[29] Xiong YS, Cheng Y, Lin QS, et al. Increased expression of Siglec-1 on peripheral blood monocytes and its role in mononuclear cell reactivity to autoantigen in rheumatoid arthritis. Rheumatology (Oxford). 2014;53(2):250–9. 24196391 10.1093/rheumatology/ket342

[30] Affandi AJ, Olesek K, Grabowska J, et al. CD169 defines activated CD14(+) monocytes with enhanced CD8(+) T cell activation capacity. Front Immunol. 2021;12: 697840. 34394090 10.3389/fimmu.2021.697840 PMC8356644

[31] Remmler J, Schneider C, Treuner-Kaueroff T, et al. Increased level of interleukin 6 associates with increased 90-day and 1-year mortality in patients with end-stage liver disease. Clin Gastroenterol Hepatol. 2018;16(5):730–7. 28919544 10.1016/j.cgh.2017.09.017

[32] Shalova IN, Lim JY, Chittezhath M, et al. Human monocytes undergo functional re-programming during sepsis mediated by hypoxia-inducible factor-1α. Immunity. 2015;42(3):484–98. 25746953 10.1016/j.immuni.2015.02.001

[33] Saha B, Bala S, Hosseini N, Kodys K, Szabo G. Krüppel-like factor 4 is a transcriptional regulator of M1/M2 macrophage polarization in alcoholic liver disease. J Leukoc Biol. 2015;97(5):963–73. 25740962 10.1189/jlb.4A1014-485R PMC6608000

[34] Liu K, Wang FS, Xu R. Neutrophils in liver diseases: pathogenesis and therapeutic targets. Cell Mol Immunol. 2021;18(1):38–44. 33159158 10.1038/s41423-020-00560-0 PMC7852892

[35] Gao B, Ahmad MF, Nagy LE, Tsukamoto H. Inflammatory pathways in alcoholic steatohepatitis. J Hepatol. 2019;70(2):249–59. 30658726 10.1016/j.jhep.2018.10.023 PMC6361545

[36] Chang B, Xu MJ, Zhou Z, et al. Short- or long-term high-fat diet feeding plus acute ethanol binge synergistically induce acute liver injury in mice: an important role for CXCL1. Hepatology. 2015;62(4):1070–85. 26033752 10.1002/hep.27921 PMC4589443

[37] Xiao L, Tang S, Zhang L, et al. Serum CXCL1 Is a prognostic factor for patients with hepatitis B virus-related acute-on-chronic liver failure. Front Med (Lausanne). 2021;8: 657076. 34395462 10.3389/fmed.2021.657076 PMC8355541

[38] Kisseleva T, Brenner D. Molecular and cellular mechanisms of liver fibrosis and its regression. Nat Rev Gastroenterol Hepatol. 2021;18(3):151–66. 33128017 10.1038/s41575-020-00372-7

[39] Tsutsui H, Nishiguchi S. Importance of Kupffer cells in the development of acute liver injuries in mice. Int J Mol Sci. 2014;15(5):7711–30. 24802875 10.3390/ijms15057711 PMC4057701

[40] Ju C, Reilly TP, Bourdi M, et al. Protective role of Kupffer cells in acetaminophen-induced hepatic injury in mice. Chem Res Toxicol. 2002;15(12):1504–13. 12482232 10.1021/tx0255976

[41] Holt MP, Yin H, Ju C. Exacerbation of acetaminophen-induced disturbances of liver sinusoidal endothelial cells in the absence of Kupffer cells in mice. Toxicol Lett. 2010;194(1–2):34–41. 20123118 10.1016/j.toxlet.2010.01.020

[42] Matsuo S, Nabekura T, Matsuda K, Shibuya K, Shibuya A. DNAM-1 Immunoreceptor Protects Mice from Concanavalin A-Induced Acute Liver Injury by Reducing Neutrophil Infiltration. *J Immunol.* 2023. 10.4049/jimmunol.2200705 37522739

[43] Marques PE, Amaral SS, Pires DA, et al. Chemokines and mitochondrial products activate neutrophils to amplify organ injury during mouse acute liver failure. Hepatology. 2012;56(5):1971–82. 22532075 10.1002/hep.25801

[44] Calvente CJ, Tameda M, Johnson CD, et al. Neutrophils contribute to spontaneous resolution of liver inflammation and fibrosis via microRNA-223. J Clin Invest. 2019;129(10):4091–109. 31295147 10.1172/JCI122258 PMC6763256

[45] Ramachandran P, Pellicoro A, Vernon MA, et al. Differential Ly-6C expression identifies the recruited macrophage phenotype, which orchestrates the regression of murine liver fibrosis. Proc Natl Acad Sci U S A. 2012;109(46):E3186-3195. 23100531 10.1073/pnas.1119964109 PMC3503234

