# Supplementary figures and images for: Elevated CD169 expressing monocyte/macrophage promotes systemic inflammation and disease progression in cirrhosis
Source: Clin Exp Med. 2024 Feb 28;24(1):45. doi: 10.1007/s10238-024-01305-3 (PMC10899294; doi:10.1007/s10238-024-01305-3)

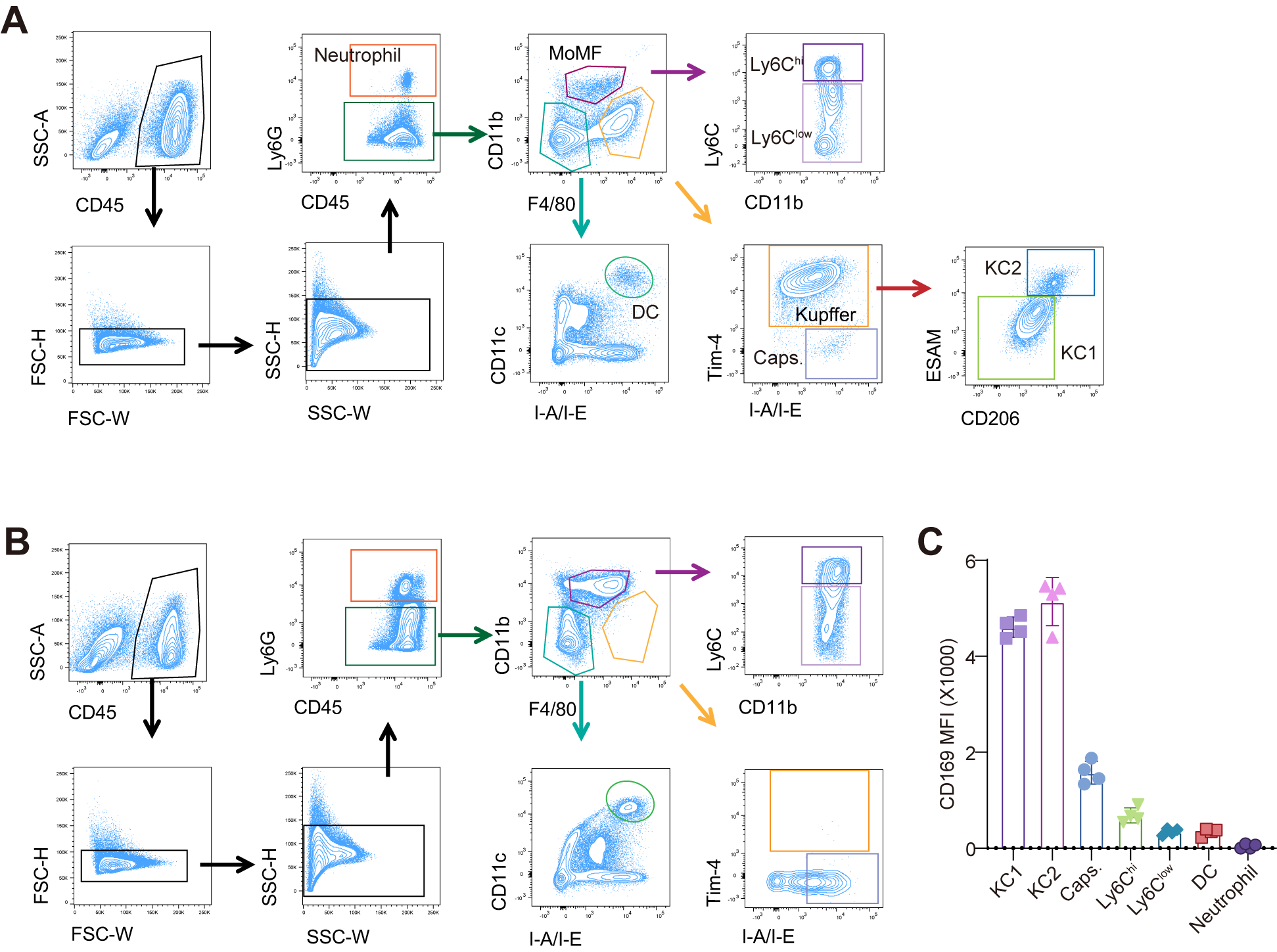

Supplement: Supplementary file 1 — (TIF 1458 KB) [file 10238_2024_1305_MOESM1_ESM.tif]

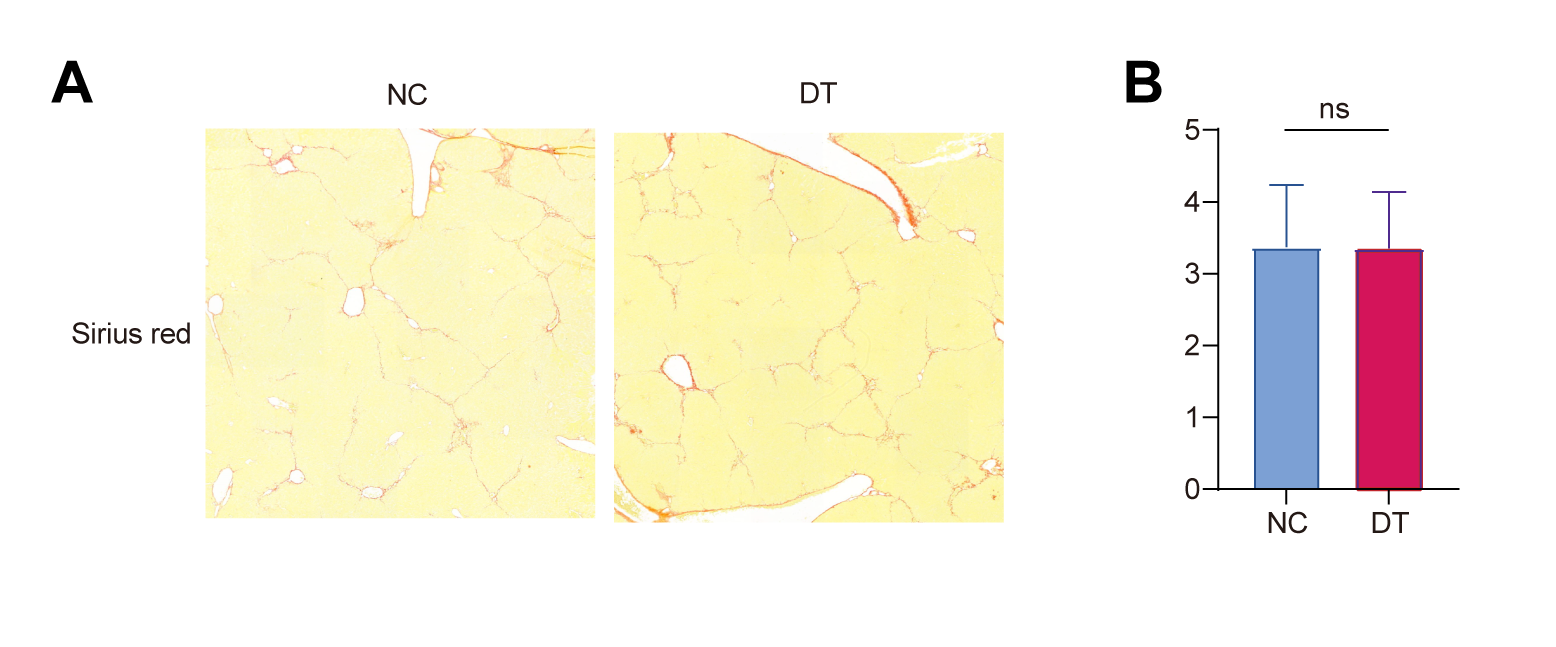

Supplement: Supplementary file 2 — (TIF 1598 KB) [file 10238_2024_1305_MOESM2_ESM.tif]
