# Supplementary material for: Elevated CD169 expressing monocyte/macrophage promotes systemic inflammation and disease progression in cirrhosis
Source: Clin Exp Med. 2024 Feb 28;24(1):45. doi: 10.1007/s10238-024-01305-3 (PMC10899294; doi:10.1007/s10238-024-01305-3)
Supplement: Supplementary file 3 — (DOCX 18 KB) [file 10238_2024_1305_MOESM3_ESM.docx]

Supplementary Table 1. The demographic and clinical characteristics of recruited subjects

| Variables | HC (n=23) |  | C-LC(n=32) | D-LC(n=40) |
| --- | --- | --- | --- | --- |
| Age(y) | 52(48-55) |  | 54(52-64) | 56(49-65) |
| Male/Female | 12/11 |  | 23/9 | 28/12 |
| Laboratory parameters | - |  |  |  |
| ALT(U/L) | - |  | 17.0(16.0-26.5) | 48.0(25.0-66.8) |
| AST(U/L) | - |  | 24.0(22.5-35.5) | 45.0(24.0-75.2) |
| TBIL(μmol/L) | - |  | 17.4(8.9-27.3) | 30.2(18.5-63.3) |
| INR | - |  | NA | 1.41(1.31-1.88) |
| Creatinine(μmol/L) | - |  | 69.1(55.0-77.5) | 68.0(49.0-89.5) |
| Decompensation events | - |  |  |  |
| Ascites (%) | - |  | 0(0) | 35(87.5) |
| UGH (%) | - |  | 0(0) | 12(30.0) |
| HE (%) | - |  | 0(0) | 4(10.0) |
| Severity score | - |  |  |  |
| MELD | - |  | - | 15.1(9.5-19.0) |

Abbreviations: HC, healthy control, C-LC, compensated liver cirrhosis, D-LC, decompensated liver cirrhosis ALT, alanine aminotransferase; AST, aspartate aminotransferase; TBIL, total bilirubin; INR, international normalized ratio; UGH, upper gastrointestinal hemorrhage; HE, hepatic encephalopathy; MELD, Model for End-Stage Liver Disease.
