# Supplementary material for: Elevated CD169 expressing monocyte/macrophage promotes systemic inflammation and disease progression in cirrhosis
Source: Clin Exp Med. 2024 Feb 28;24(1):45. doi: 10.1007/s10238-024-01305-3 (PMC10899294; doi:10.1007/s10238-024-01305-3)
Supplement: Supplementary file 4 — (DOC 43 KB) [file 10238_2024_1305_MOESM4_ESM.doc]

Supplementary Table 1. Antibodies

| **Antibodies and reagents** | **clone** | **Manufacturers** |
| --- | --- | --- |
| PE anti-human CD169 | 7-239 | Biolegend |
| Percp-cy5.5 anti-human CD16 | 3G8 | Biolegend |
| FITC anti-human CD14 | HCD14 | Biolegend |
| Percp-cy5.5 anti-human HLA-DR | L243 | Biolegend |
| PE-CF594 anti-human CD64 | 10.1 | BD bioscience |
| BV421 anti-human CD206 | 19.2 | BD bioscience |
| BV605 anti-human CD80 | L307.4 | BD bioscience |
| BB700 anti-human CD80 | MEM-233 | ebioscience |
| Percp-cy5.5 anti-human CCR5 | J418F1 | Biolegend |
| PE anti-human CX3CR1 | 2A9-1 | BD bioscience |
| APC anti-human CCR2 | K036C2 | Biolegend |
| BV510 anti-human CCR7 | 3D12 | BD bioscience |
| APC anti-human CD14 | M5E2 | BD bioscience |
| PE anti-mouse CD169 | 3D6.112 | Biolegend |
| Percp-cy5.5 anti-mouse MHC II | M5/114.15.2 | Biolegend |
| AF700 anti-mouse/human CD11b | M1/70 | Biolegend |
| BV421 anti-mouse Ly-6C | HK1.4 | Biolegend |
| PE-Cy7 anti-mouse Tim-4 | RMT4-54 | Biolegend |
| BV785 anti-mouse CD206 (MMR) | C068C2 | Biolegend |
| APC anti-mouse ESAM | 1G8/ESAM | Biolegend |
| FITC anti-mouse CD11c | N418 | Biolegend |
| FITC anti-mouse CD45 | I3/2.3 | Biolegend |
| BV605 anti-mouse Ly6G | 1A8 | Biolegend |
| BUV395 anti-mouse F4/80 | T45-2342 | BD bioscience |
